# Therapeutic effect of localized vibration on alveolar bone of osteoporotic rats

**DOI:** 10.1371/journal.pone.0211004

**Published:** 2019-01-29

**Authors:** Mani Alikhani, Mona Alikhani, Sarah Alansari, Abdullah Almansour, Mohammad A. Hamidaddin, Edmund Khoo, Jose A. Lopez, Jeanne M. Nervina, Joo Y. Nho, Serafim M. Oliveira, Chinapa Sangsuwon, Cristina C. Teixeira

**Affiliations:** 1 Advanced Graduate Education Program in Orthodontics, Department of Developmental Biology, Harvard School of Dental Medicine, Boston, Massachusetts, United States of America; 2 The Forsyth Institute, Cambridge, Massachusetts, United States of America; 3 CTOR Academy, Hoboken, New Jersey, United States of America; 4 Department of Orthodontics, New York University College of Dentistry, New York, New York, United States of America; 5 Department of Mechanical Engineering, Polytechnic Institute of Viseu, Viseu, Portugal; 6 Department of Basic Science & Craniofacial Biology, New York University College of Dentistry, New York, New York, United States of America; VA Loma Linda Healthcare System, UNITED STATES

## Abstract

**Objectives:**

Vibration, in the form of high frequency acceleration (HFA), stimulates alveolar bone formation under physiologic conditions and during healing after dental extractions. It is not known if HFA has an anabolic effect on osteoporotic alveolar bone. Our objective is to determine if HFA has a regenerative effect on osteoporotic alveolar bone.

**Methods and materials:**

Adult female Sprague-Dawley rats were divided into five groups: 1) Ovariectomized Group (OVX), 2) Sham-OVX Group that received surgery without ovariectomy, 3) OVX-HFA Group that was ovariectomized and treated daily with HFA, 4) OVX+Static Force Group that was ovariectomized and received the same force as HFA, but without vibration, and 5) Control Group that did not receive any treatment. All animals were fed a low mineral diet for 3 months. Osteoporosis was confirmed by micro-CT of the fifth lumbar vertebra and femoral head. HFA was applied to the maxillary first molar for 5 minutes/day for 28 and 56 days. Maxillae were collected for micro-CT, histology, fluorescent microscopy, protein and RNA analysis, and three-point bending mechanical testing.

**Results:**

Micro-CT analysis revealed significant alveolar bone osteoporosis in the OVX group. Vibration restored the quality and quantity of alveolar bone to levels similar to the Sham-OVX group. Animals exposed to HFA demonstrated higher osteoblast activity and lower osteoclast activity. Osteogenic transcription factors (RUNX2, Foxo1, Osterix and Wnt signaling factors) were upregulated following vibration, while RANKL/RANK and Sclerostin were downregulated. HFA did not affect serum TRAcP-5b or CTx-1 levels. The osteogenic effect was highest at the point of HFA application and extended along the hemimaxillae this effect did not cross to the contra-lateral side.

**Conclusions:**

Local application of vibration generated gradients of increased anabolic metabolism and decreased catabolic metabolism in alveolar bone of osteoporotic rats. Our findings suggest that HFA could be a predictable treatment for diminished alveolar bone levels in osteoporosis patients.

## Introduction

Osteoporosis is a silent and progressive disease that is characterized by reduced bone mineral density, altered protein composition and continuous deterioration of bone microarchitecture [1]. Osteoporosis can occur in both women and men; however, it is well documented that women are more often diagnosed with osteoporosis compared to men, primarily due to the sudden decline in estrogen during menopause [2].

In osteoporosis, the normal balance between bone resorbing cells (osteoclasts) and bone forming cells (osteocyte and osteoblasts) is altered [3]. The result of this imbalance is an increase in bone fragility and susceptibility to fracture, especially in the spine, hip and distal forearm [4]. However, risk associated with osteoporosis is not limited to weight-bearing bones. For example, osteoporosis can severely impact oral health by reducing the health of alveolar bone leading to tooth loss [5–7]. It has been suggested that women with osteoporosis are three times more likely to experience tooth loss than those who do not have the disease [8]. Because tooth retention and a functional dentition are key determinants of nutritional status, tooth loss due to osteoporosis of alveolar bone may predispose the patients not only to other chronic diseases, but can worsen the existing osteoporosis through a negative feedback loop [9]. In addition to tooth loss, osteoporosis is positively correlated with increased periodontal disease occurrence and progression [10] and decreased dental implant integration and stability [11].

Due to the morbidity associated with osteoporosis of weight bearing bones, different therapeutic approaches to prevent bone loss or to regenerate lost bone have been suggested. From these approaches, pharmacological agents, such as bisphosphonates, are the most commonly used to prevent bone loss. Unfortunately, long- term usage of these drugs is associated with many adverse side effects, especially bony lesions in the oral cavity such as osteonecrosis [1, 12]. Due to these side effects, non-pharmacological approaches, such as change in life style, nutritional support and especially mechanical stimulation, are gaining increased attention and popularity [13].

As a mechano-sensitive tissue, bone adapts its mass, microstructure, and strength in response to dynamic mechanical loading. Hence, people with osteoporosis are encouraged to exercise their musculoskeletal system through moderate-to-high-intensity weight-bearing physical activity to increase bone mineral density (BMD) [14]. However, the elderly are often not able to undertake these exercises at a level needed to show improvement. One possible alternative modality that mimics physical activity is low- magnitude, low-impact, high-frequency whole body vibration [15–17].

While mechanical stimulation in the form of whole-body vibration has demonstrated promising results in the treatment of osteoporosis in weight bearing bones, its effect on non-weight bearing bones, such as the craniofacial skeleton, has not been very successful. This may be due to two facts related to the experimental exposure to vibration: 1) subjects need to stand on vibrating platforms, which exposes primarily the weight bearing bones, rather than the craniofacial skeleton, to the stimulation, and 2) soft tissue may attenuate the effect of whole-body vibration on other bones, especially in the craniofacial skeleton. However, there is significant evidence that non-weight bearing bones, especially alveolar bone, is also sensitive to dynamic mechanical stimulation. First, as in weight-bearing bones, mechanical stimulation plays a significant role in the health of alveolar bone [18]. The replacement of a regular diet with a soft diet [19] or the lack of function due to missing teeth is accompanied by a significant decrease in alveolar bone density [20]. Second, we have recently discovered that applying vibration with a specific acceleration and frequency (120Hz, peak acceleration of 0.3g and peak strain of 10 *με*) directly on teeth, in the absence of significant load, has an osteogenic effect on healthy alveolar bone and also preserves alveolar bone structure after tooth extraction [21, 22]. It should be emphasized that contrary to weight bearing bones, loading of alveolar bone is indirect via teeth, and due to their delicate structure, application of high load in the oral cavity is not possible. Therefore, increasing the frequency and acceleration of the applied load compensates for the impossibility of applying high magnitude loads inside the mouth. However, it is not clear if this mechanical stimulation can reverse the negative structural changes in alveolar bone due to osteoporosis.

In this study, we hypothesized that low magnitude force and vibration, in the form of HFA, may produce osteogenesis in osteoporotic alveolar bone, similar to what is observed in healthy alveolar bone.

## Materials and methods

### Animal model, study design and surgery

**Fig 1:**
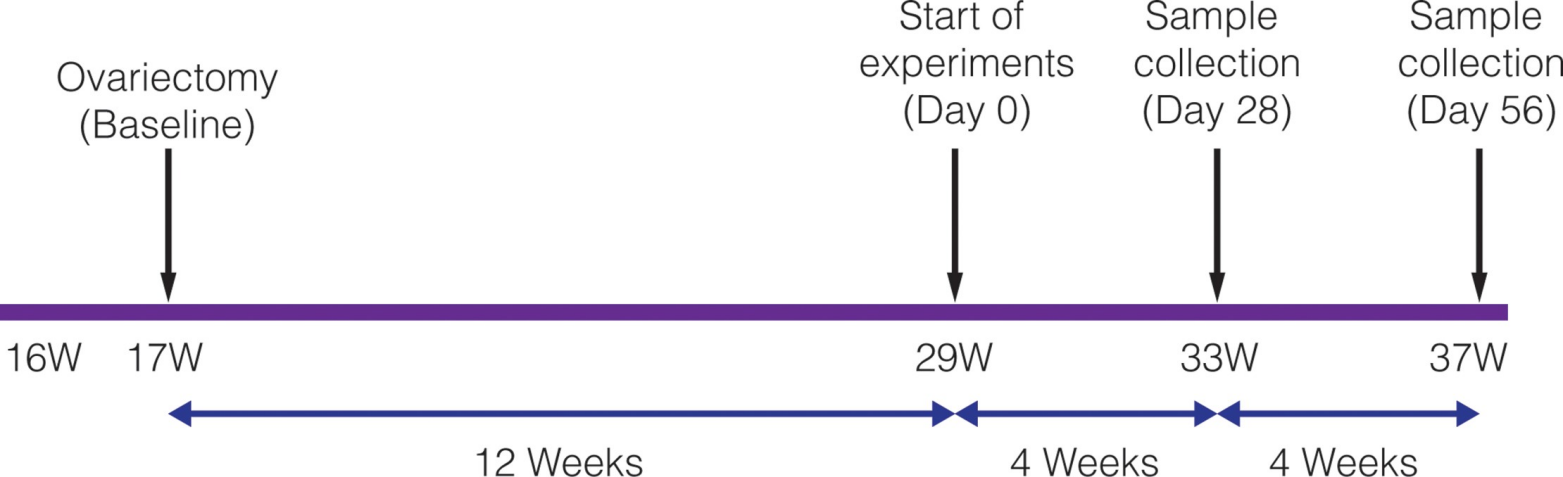
Study time line. Schematic of study time line. At 17 weeks, animals were ovariectomized and fed a low mineral diet for 12 weeks. Study of the HFA effect was then initiated and samples were collected 4 and 8 weeks later, at 33 and 37 weeks, respectively.

Adult female Sprague-Dawley rats (n = 114, average weight 220–245 g, 16 weeks old) were housed and treated according to a protocol conforming to ARRIVE (Animal Research Reporting of the In Vivo Experiments) guidelines and approved by the New York University Institutional Animal Care and Use Committee. The timeline of the experiment is detailed in Fig 1. After one week of arrival at NYU, animals were divided into five groups: 1) Control Group that did not receive any intervention; 2) OVX Group that received bilateral ovariectomy according to FDA guidelines (see protocol below) 3) Sham Group that received ovariectomy surgery without having the ovaries removed; 4) OVX+HFA Group that received ovariectomy and HFA (frequency of 120 Hz and peak acceleration of 0.3g where ‘g’ represents the acceleration of the earth (1g = 9.81 m/s^2^)) for 5 minutes per day; and 5) OVX+Static Group that received 10 μ*ε* of static load for 5 minutes per day.

For ovariectomy, briefly a small dorsal midline incision was made. The abdominal cavity was entered via a blunt puncture through the abdominal wall. The ovary (and attached fat pad) was pulled out the abdominal cavity and the fallopian tube was cauterized and the ovary was dissected. The skin incision was closed with wound clips.

All animals were housed under the same conditions and were fed a low mineral diet ((calcium (1.25%), phosphorus (0.875%), magnesium (0.084%), zinc (approximately 0.006%), and fluorine (approximately 0.001%), components of AIN-93M (3.5% minerals) were decreased to less than 0.001% and replaced with sucrose) for 3 months (S1 and S2 Tables). All animals survived and were in good condition during the entire experimental period. Osteoporosis was confirmed with microCT (μCT) analysis of the fifth lumbar vertebral body (L5) and femoral heads.

### High frequency acceleration device

The high frequency acceleration (HFA) device [21, 22] was prepared and calibrated to deliver a total resultant of 0.3g gravitational acceleration (combining all three accelerations with the mean peak acceleration in medial-lateral (0.1g), antero-posterior (0.3g), and vertical (0.2g)) at 120Hz frequency with total displacement of 20μm (Mechanical Engineering Department of the Polytechnic Institute of Viseu-Portugal). Appliance calibration was performed with a sensor (OMRON-E2E-X7D1-N 23304; OMRON Electronics Iberia SAU, Lisbon, Portugal) that was connected to an oscilloscope (Metrix OX 803B 40 MHz, Metrix Electronics, Hampshire, United Kingdom) and a Digital Tachometer (Lutron DT 2236, Lutron Electronic Enterprise, Taipei, Taiwan). Strain gauges (UFLK-1-11-1L, 1 mm gauge length, 120 Ω TML Gages, Texas Measurements, College Station, TX, USA) were attached using cyanoacrylate to the palatal and buccal sides of the alveolar bone near the maxillary first molar on fresh and dry rat skulls. Strain signals were amplified by a low- noise amplifier (SX500, Beacon Dynamics, Byram Township, NJ, USA). Data acquisition and analyses were performed with the SPIDER 8 system and Catman 4.5 software measured with a piezoelectric sensor-Bimorph vibration element 4V5mm (Allied Electronics, Fort Worth, TX, USA), and a MotionNode 3-DOF inertial measurement unit (GLI Interactive, Seattle, WA, USA). Vibration produce a very low peak strain of 10 *με*) microstrain), measured at the buccal alveolar bone surrounding the maxillary first molar.

### HFA and static force application

The HFA regimen and static force were applied to the maxillary right first molar and produced an average peak strain of 10 μ*ε* in the surrounding bone, as measured by a strain gauge. All vibration stimulation was applied to the occlusal surface of the upper first maxillary molar for 5 minutes per day for 28 and 56 days under 3% isoflurane anesthesia. For some animals, bone labeling was performed with demeclocycline (i.p. injection; 50mg/kg) at Days 0 and 54 and calcein green (i.p. injection; 15mg/kg) at Day 28. Animals were sacrificed at Days 0, 28 and 56 after HFA application by CO_2_ narcosis, and the maxillae were collected for μCT analysis, fluorescence microscopy, histology, RNA and protein analysis and mechanical testing.

### Micro-CT imaging

**Fig 2:**
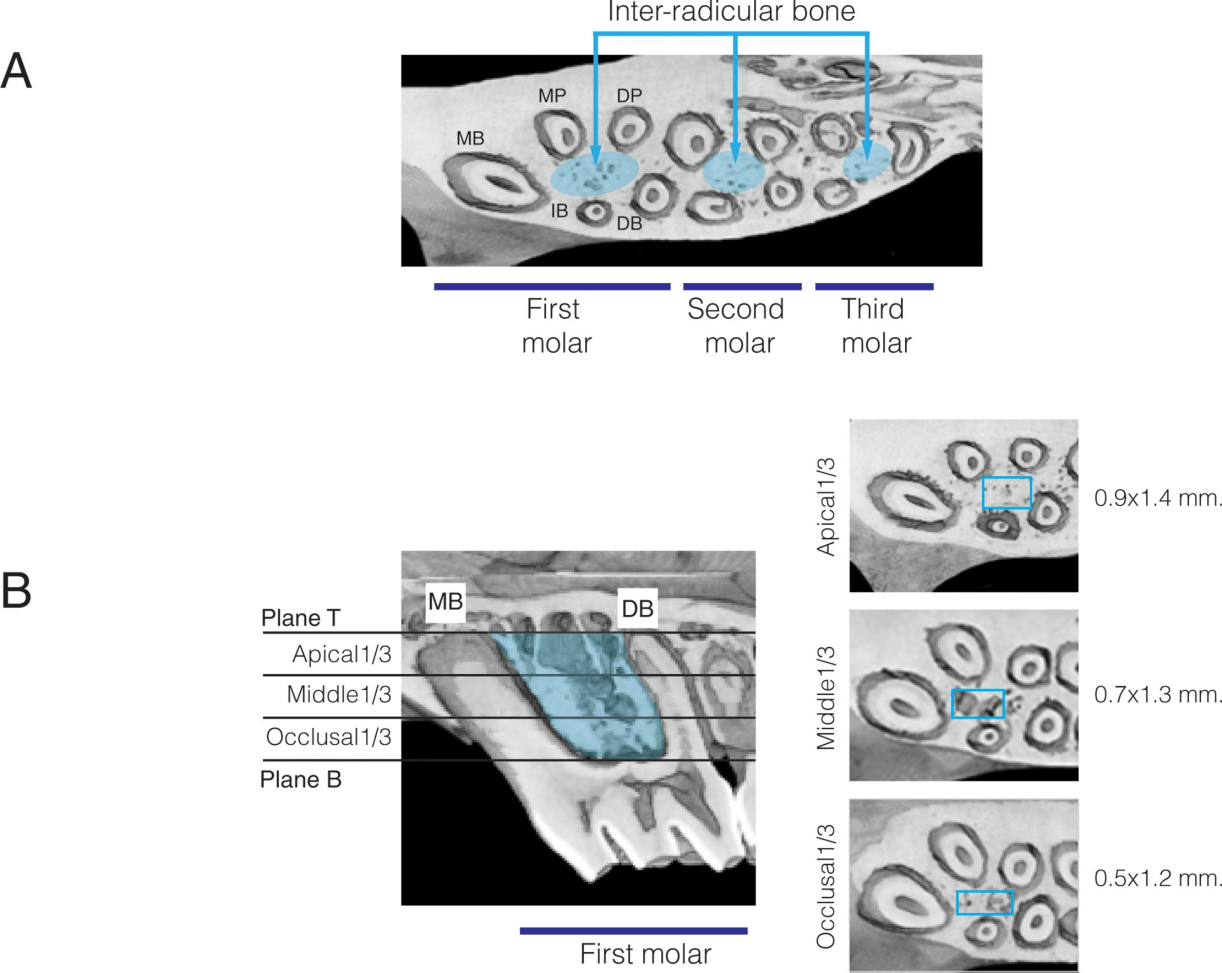
Region of interest (ROI). **(A)** Inter-radicular bone area used for bone quantity and quality measurements (MB, mesial-buccal root; IB, intermediate buccal root; DB, disto-buccal root; MP, mesial-palatine root; DP, disto-palatine root). **(B)** ROI in the area of the maxillary first molar is limited from below with a horizontal plane parallel to the occlusal plane (Plane B) passing through the alveolar ridge crest; from the top by a horizontal plane passing through the apex of the mesio-buccal root and disto-buccal root (Plane T). The area between Planes B and T was divided equally into occlusal, middle and apical thirds to measure bone quantity and quality. The rectangular boxes have different dimensions, increasing in size apically as the roots diverge.

Maxillae were scanned in a Scanco MicroCT (μCT40, Scanco Medical AG, Bassersdorf, Switzerland). The bones were scanned at an energy of 70kV and intensity of 114 μA, with 300 ms integration time, resulting in 16 μm isotropic voxel size. Results were analyzed utilizing μCT V6.0 software on the HP open platform (OpenVMS Alpha Version 1.3–1 session manager). The scanned area of interest included the three maxillary molars (Fig 2A). The Region of Interest (ROI) focused on the inter-radicular bone of the first, second and third molars. The area of inter-radicular bone of the first molar (Fig 2B) was delimited by the following planes: 1) Superior plane: A horizontal plane parallel to the occlusal plane passing through the alveolar crest; 2) Inferior plane: A horizontal plane passing through the apex of mesio-buccal root; 3) Mesial plane: A vertical plane tangent to the mesial surface of the mesio-buccal root; 4) Distal plane: A vertical plane tangent to the distal surface of the disto-buccal and disto-palatal roots; 5) Buccal plane: A vertical plane tangent to the buccal surface of the mesial and disto-buccal roots; 6) Palatal plane: A vertical plane tangent to the palatal surface of the mesial and disto-palatal roots. This ROI was further divided vertically into equal thirds. In each third, bone parameters were measured in a rectangular area that was defined based on the apical divergence of the roots. The area in the first third started from the furcation and continued apically for 40 slices (approximately 0.5 x 1.2 mm). In the middle and apical thirds the rectangular dimension was increased to 0.7 x 1.3 mm and 0.9 x 1.4 mm, respectively, and each extended for another 40 slices. The bone density was defined by morphing across the slices in each area. Trabecular bone volume fraction (BV/TV%), trabecular thicknesses (Tb. Th.), trabecular number (Tb. N.), and trabecular space (Tb. Sp.) were determined for each third.

### Histology and fluorescence microscopy

Hemi-maxillae were fixed in 4% paraformaldehyde, demineralized in 14% EDTA solution for 2 weeks, dehydrated in alcohol series, embedded in paraffin, and cut into 5 μm occlusal sections. Five sections through the middle third were stained with hematoxylin and eosin (H&E). Intermediate sections were immunostained (Vectastain ABC kit; Vector Laboratories, Burlingame, CA, USA) with an antibody for tartrate-resistant acid phosphatase (TRAcP-5b; Zymed antibodies; Invitrogen, Carlsbad, CA, USA), a marker of osteoclasts. As a negative control, sections were exposed to pre-immune serum. Sections were scanned on a Scan Scope GL optical microscope (Aperio, Bristol, UK) at 20x magnification. Osteoclasts were defined as TRAcP-5b–positive multinuclear cells on periosteal and endosteal bone surfaces of inter-radicular bone of first molars. The number of TRAcP-5b positive cells per 1 mm^2^ of alveolar bone was determined in 5 consecutive sections from the middle third and averaged. Two examiners completed all histological quantifications.

For fluorescence microscopy, specimens were fixed in formalin, washed overnight in running water, dehydrated in an alcohol series, cleared with xylene, and embedded in methyl methacrylate. Samples were sectioned at 5-μm thickness on a RM 2265 Leica microtome (Leica Biosystems, Buffalo Grove, IL, USA), viewed and photographed (Leica DMRX/E Universal Microscopy, Turboscan Software, Cambridge, UK).

### RNA analysis

For total RNA extraction, five animals from each group were sacrificed by CO_2_ narcosis at 24 hours, and the hemi-maxillae were dissected and frozen in liquid nitrogen. Total RNA was collected as described previously [23]. Real-time PCR for bone formation and bone resorption markers was performed with primers specific for rat genes, with a QuantiTect SYBR Green RT-PCR kit (Qiagen, Valencia, CA) on a DNA Engine Optican 2 System (MJ Research, Waltham, MA). An mRNA pool for each group was tested three times. Relative levels of mRNA were calculated and normalized to the level of GAPDH and acidic ribosomal protein mRNA.

### Bone activity marker analysis

Enzyme-linked immunosorbent assay (ELISA) kits were used to measure serum Tartrate-resistant acid phosphatase-5b (TRAcP-5b) (Kamiya Biochemical Company, Seattle, WA) and C-terminal cross-linked telopeptides of type I collagen (CTx-1) (Immunodiagnostic Systems, Fountain Hills, AZ) according to the manufacturer’s instructions.

### Three-point bending test

**Fig 3:**
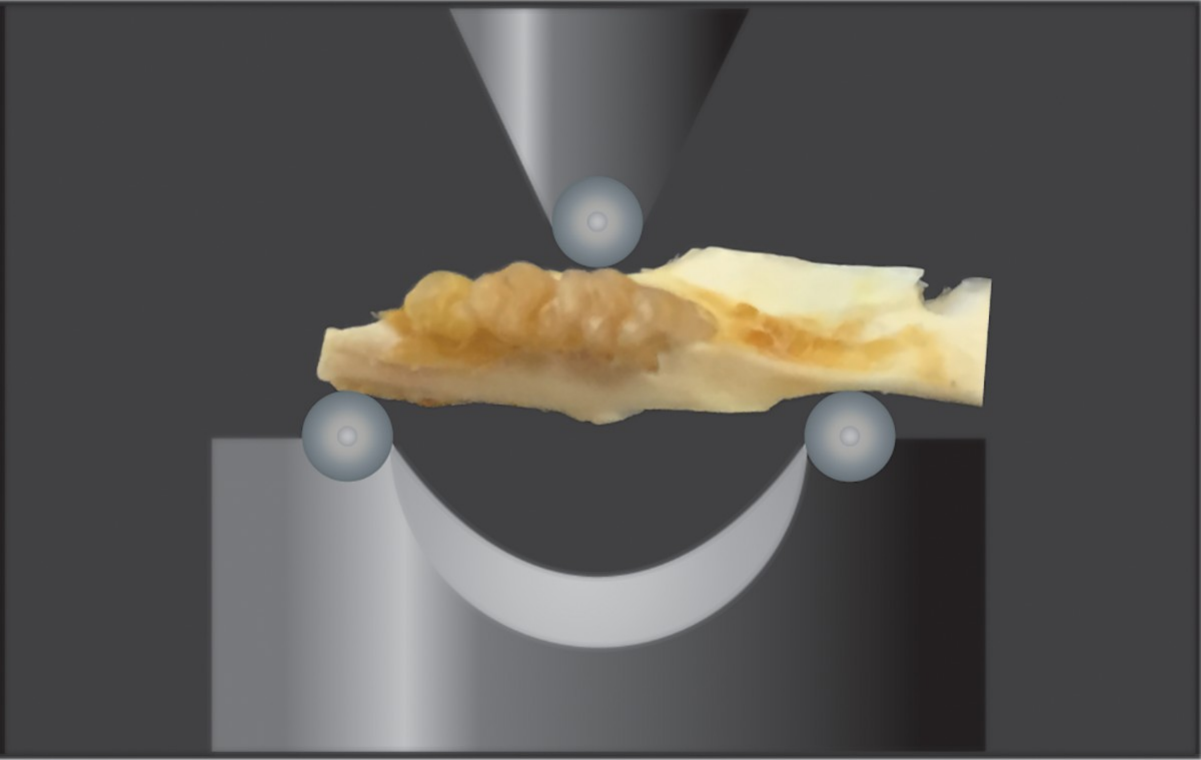
Mechanical testing of hemimaxilla. Schematic view of three-point bending test of the rat hemimaxilla. Hemi-maxillae were placed buccal-side upward. The central loading point was aligned at the first molar midpoint. Maximum load, yield load and stiffness were computed at a displacement rate of 1mm/min.

Hemi-maxillae were separated from the basisphenoid, zygomatic, frontal and premaxilla bones. Biomechanical properties were assessed using an Instron 4206–006 universal machine equipped with a load cell (model UK1899-1kN). The hemi-maxillae were placed buccal side upward (Fig 3). The central loading point was aligned at the first molar midpoint. During testing the maxillary incisor was not part the specimen (was removed, along with the premaxilla). Three-point bending tests were performed at a displacement rate of 1mm/min and load and displacement values were recorded. Recorded data were processed and maximum load, yield load and stiffness were computed.

### Statistical analysis

After confirming normal distribution of samples by the Shapiro-Wilk test, group comparisons were assessed by analysis of variance (ANOVA). Pairwise multiple comparison analysis was performed with the Tukey’s *post hoc* test. Two-tailed *p* values were calculated; *p* < 0.05 was set as the level of statistical significance.

## Results

### Ovariectomy induced significant bone loss in alveolar bone

Development of osteoporosis was evaluated in adult animals subjected to ovariectomy or sham surgery (at week 17) and exposed to a low mineral diet for 12 weeks (Fig 1). During this experimental period, animals in all groups were healthy and did not show distress or change in activity. However, ovariectomy resulted in weight gain (Table 1).

**Table 1:** Effect of ovariectomy on body weight.

	Sham	OVX	OVX+HFA	*p value (OVX)	*p value (OVX+HFA)
**29 Weeks** **(Day 0)**	259 ± 25	343 ± 27 *	355 ± 24*	p<0.045	p<0.021
**33 Weeks** **(Day 28)**	266 ± 22	364 ± 27 *	351 ± 22*	p<0.084	p<0.021
**37 weeks** **(Day 56)**	281 ± 26	383 ± 28 *	378 ± 29*	p<0.023	p<0.032

Data demonstrate significantly higher body weights in the OVX group at all time points. (Each value represents the mean weight (g) ± SEM of 6 samples in each group, * significantly different from the Sham group at the same time point. p<0.05).

**Fig 4:**
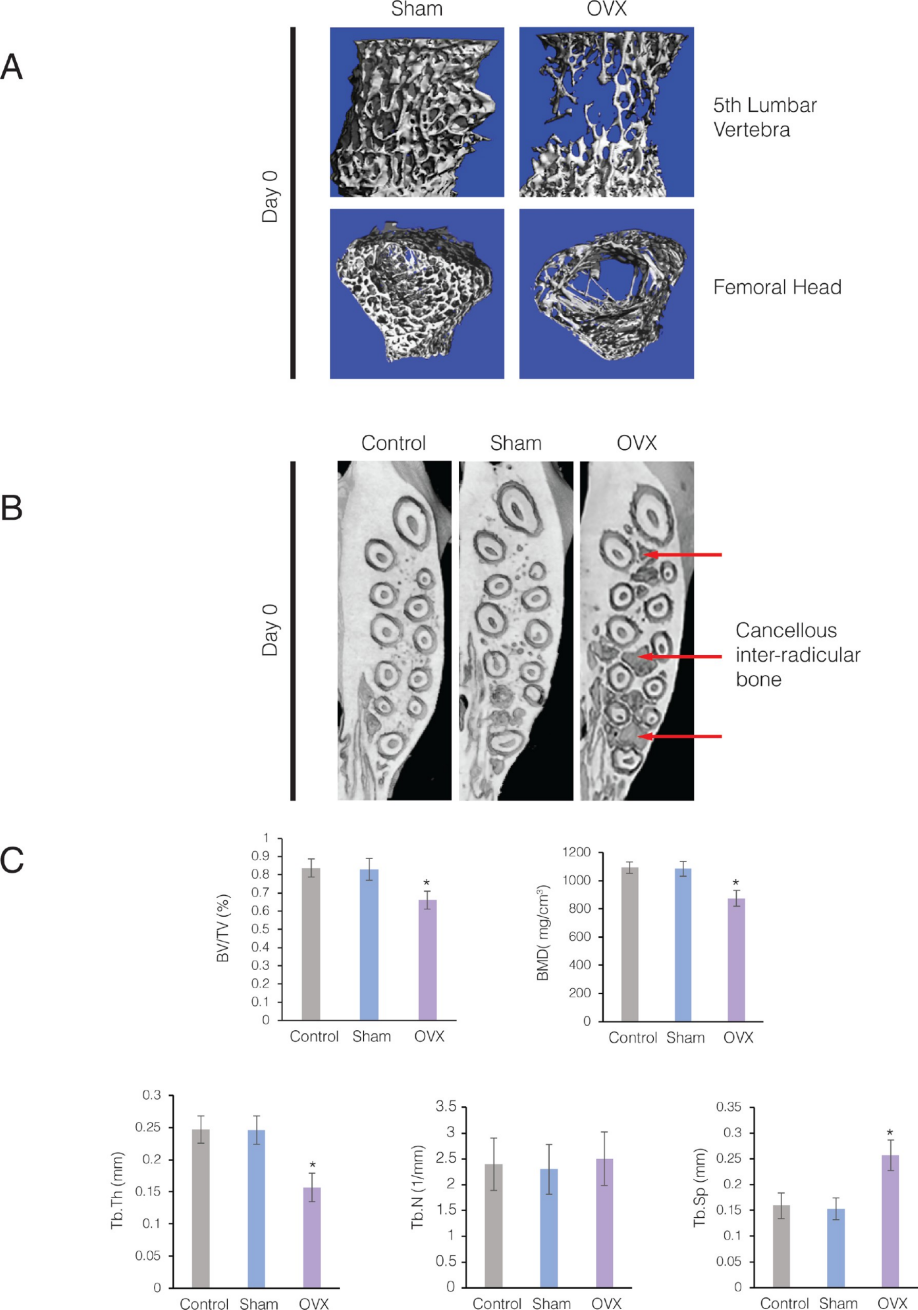
Ovariectomized rats on low mineral diet developed osteoporotic alveolar bone. **(A)** 3D μCT images of the fifth lumbar vertebral bodies and femoral head from Control and OVX rats at 29 weeks (Day 0). Note the marked loss of trabeculae in the cancellous bone of OVX animals. **(B)** Axial sections of 3D μCT scans through the maxillary alveolar bone from Control, Sham-ovariectomized (Sham), and ovariectomized rats (OVX) at 29 weeks (Day 0). Arrows point to loss of cancellous bone in the maxillary alveolar bone of the OVX animals. **(C)** Parametric values for intra-radicular bone of the maxillary first molar at Day 28. Each value represents the mean ± SEM of 6 samples.

While just prior to ovariectomy (Baseline) there were no significant differences in weight between Sham and OVX groups, at Day 0 (12 weeks after ovariectomy), Day 28 (16 weeks after ovariectomy) and Day 56 (20 weeks after ovariectomy), the weight of the OVX group was statistically higher compared to the Sham group (p<0.05). In addition, at Day 0 (12 weeks after ovariectomy), there was significant bone loss in the lumbar spine and femoral head in the OVX group compared to the Sham group (Fig 4A).

Bone loss was not limited to the spine and long bones, but the cancellous maxillary alveolar bone in OVX group also showed significant bone loss compared to the Control and Sham groups at Day 0 (Fig 4B). At Day 28, inter-radicular bone of the maxillary first molar in OVX rats demonstrated a significant decrease in bone volume fraction (BV/TV) (p<0.016), trabecular thickness (Tb. Th.) (p<0.005) and bone mineral density (BMD) (p<0.005) compared to the Control and Sham groups (Fig 4C). No significant difference was observed between Control and Sham groups for any of these parameters (p>0.05). Trabecular spacing (Tb. Sp.) significantly increased in maxillae of the OVX group compared to the maxillae of Control and Sham groups (p<0.01). However, trabecular number (Tb. N.) did not show any significant difference between Control, Sham and OVX groups. Bone volume fraction (BV/TV) of the OVX group was significantly lower compared to the Sham group at both Day 28 and Day 56. In addition, there was no difference in BV/TV within the OVX group at Day 28 and Day 56 (Fig 4D).

### High-frequency acceleration restores alveolar bone density in osteoporotic rats

**Fig 5:**
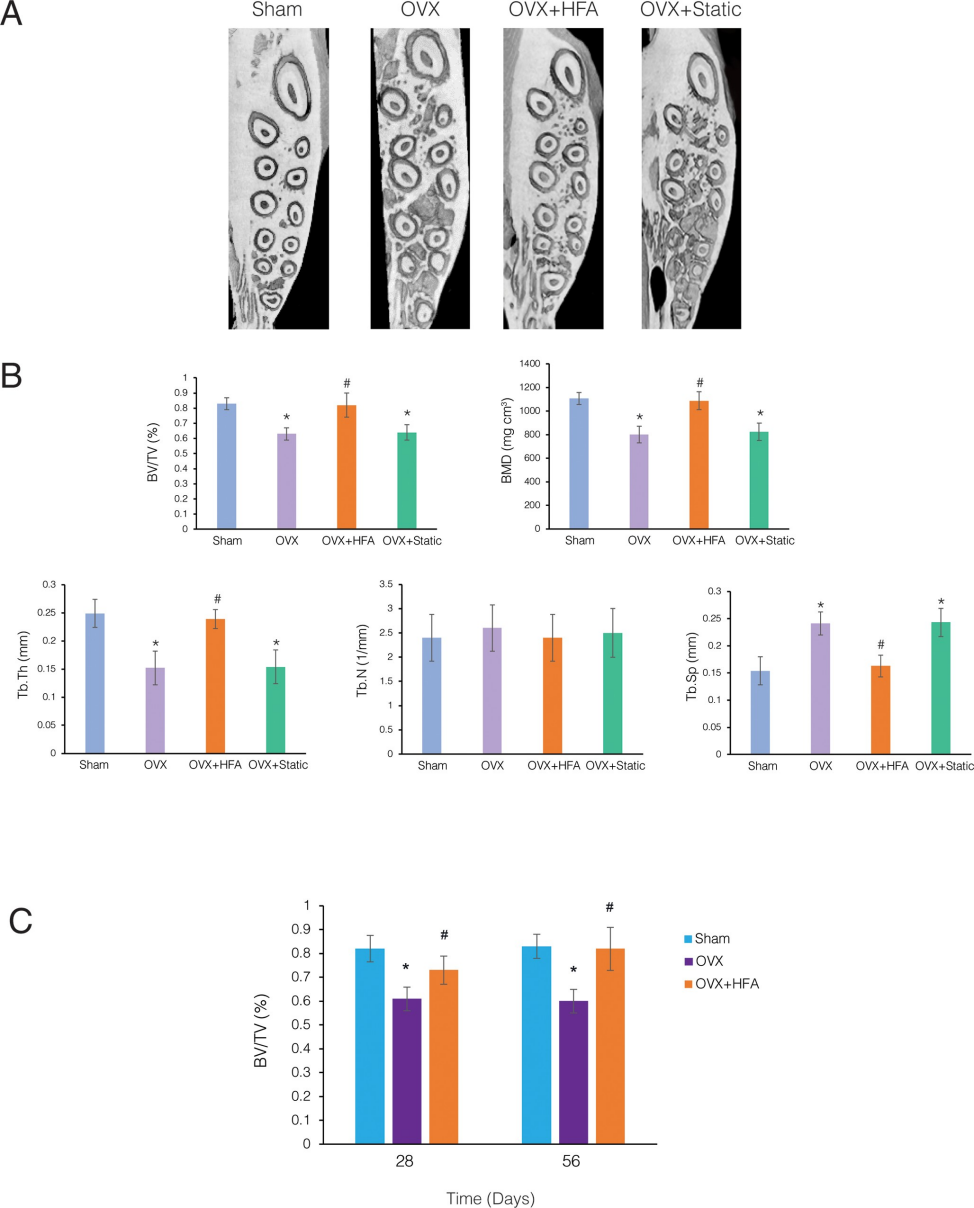
HFA reestablished the density of osteoporotic alveolar bone in rats. **(A)** Axial sections of 3D μCT images through the maxillary alveolar bone at Day 56 (20 weeks after ovariectomy) from Sham, OVX, OVX + HFA, OVX + Static groups. Significant osteoporotic changes can be appreciated in the OVX and OVX + Static groups compared to the Sham and OVX + HFA groups. **(B)** Parametric values for inter-radicular bone of the maxillary first molar at Day 56. Each value represents the mean ± SEM of 6 samples. (**C**) Comparison of BV/TV of different groups at Day 28 and Day 56. Each value represents the mean ± SEM of 6 samples (* significantly different from Sham, # significantly different from OVX group).

Application of low magnitude dynamic load (10 *με*) in the form of high frequency acceleration (HFA, 120 Hz, 0.3 peak resultant acceleration, 20 μm displacement) to the maxillary first molar for 56 days, 5 minutes per day reestablished the alveolar bone density in the maxillae of the OVX group to the levels of the Sham group as observed in the μCT scans of maxillae (Fig 5A). Interestingly, the same magnitude of static load (10 *με*) did not affect bone density of osteoporotic alveolar bone.

A quantitative evaluation of alveolar bone parameters (Fig 5B) demonstrated that HFA significantly increased BV/TV (p<0.04) and BMD (p<0.03) in OVX+HFA animals compared to OVX animals at Day 56. No significant difference was observed between OVX+HFA and Sham groups (p<0.05). This recovery was mostly through the increase in trabecular thickness (p<0.02) and decrease in trabecular spacing (p<0.015), while trabecular number remained the same (p>0.05). Interestingly, the application of a similar magnitude of static load (OVX + Static) did not cause any significant change in bone parameters when compared to the OVX group (p<0.05). Alveolar BV/TV in OVX+HFA maxillae was significantly higher than in the OVX group at both Day 28 (p<0.042) and Day 56 (p<0.002) (Fig 5C). While longer application of HFA showed higher BV/TV values, the difference between Day 28 and Day 56 was not statistically significant in OVX+HFA animals (p>0.05).

It should be emphasized that local application of HFA on the maxillary first molar did not have any effect on the weight of the animals and no difference between the weight of OVX and OVX+HFA was observed (Table 1).

### High-frequency acceleration induced expression of osteogenic markers and inhibits expression of osteoclastic markers

**Fig 6:**
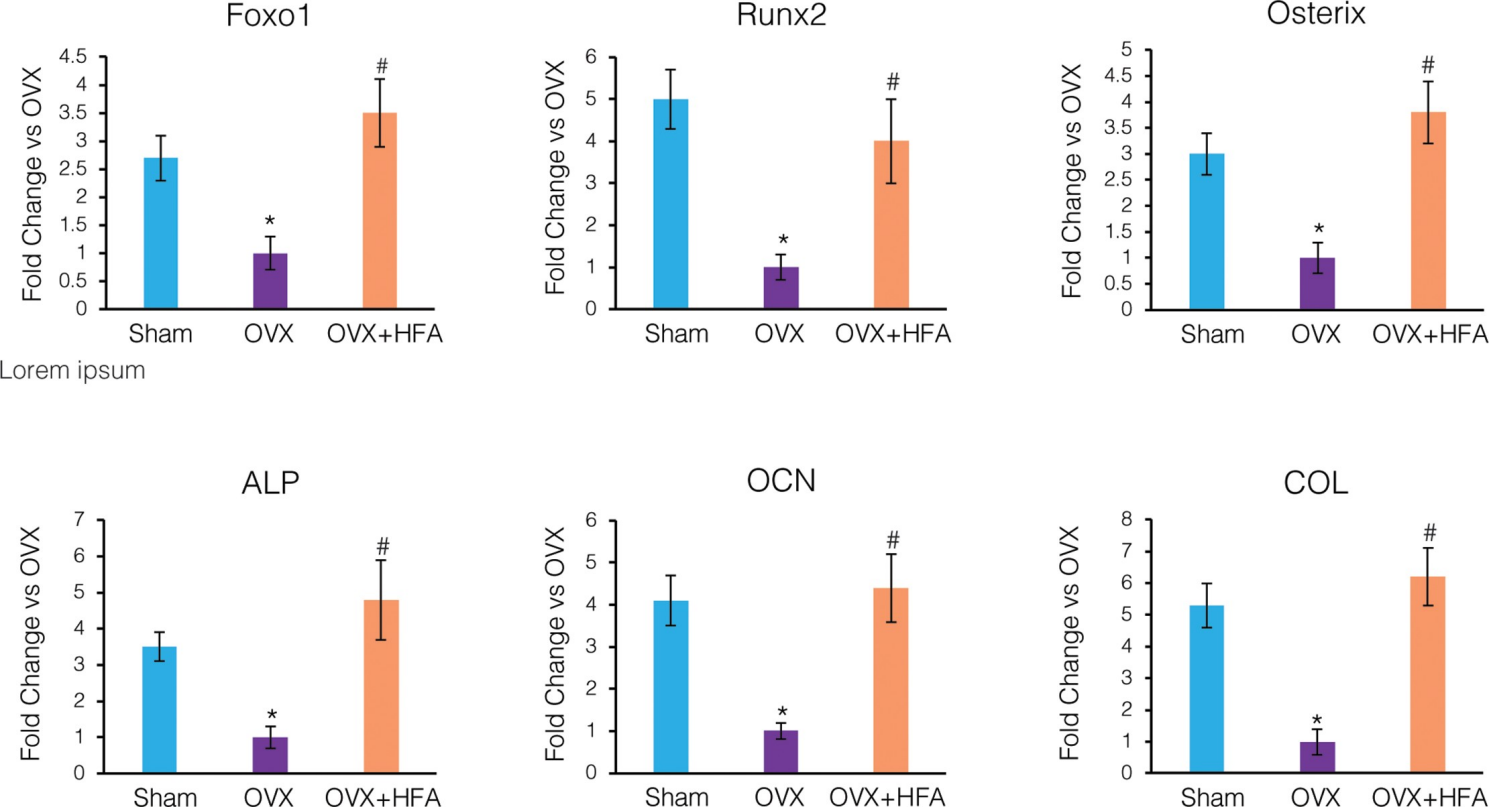
HFA increased expression of osteogenic markers in alveolar bone. Gene expression of osteogenic markers (Foxo1, Runx2, Osterix, alkaline phosphatase (ALP), osteocalcin (OCN), collagen I*α*1 (COL)) were measured by real time RT-PCR. Data shows mean fold change in mRNA levels compared to OVX, 56 days after application of HFA to the maxillary right first molar. Each value represents the mean ± SEM of 6 animals (* Significantly different from Sham group; ^#^ significantly different from OVX group).

RNA analysis demonstrated that the expression of osteogenic transcription factors Foxo1, Runx2, and Osterix significantly decreased in the OVX group compared to the Sham group at Day 28 (Fig 6). Similarly, the expression of osteogenic biomarkers ALP, OCN, ColI*α*1 was down-regulated in the OVX group (p<0.05). HFA restored the expression of these markers to levels similar to that of the Sham group (p<0.05). Indeed, no statistical difference between OVX+HFA and Sham groups was observed for any of these markers (p>0.05).

**Fig 7:**
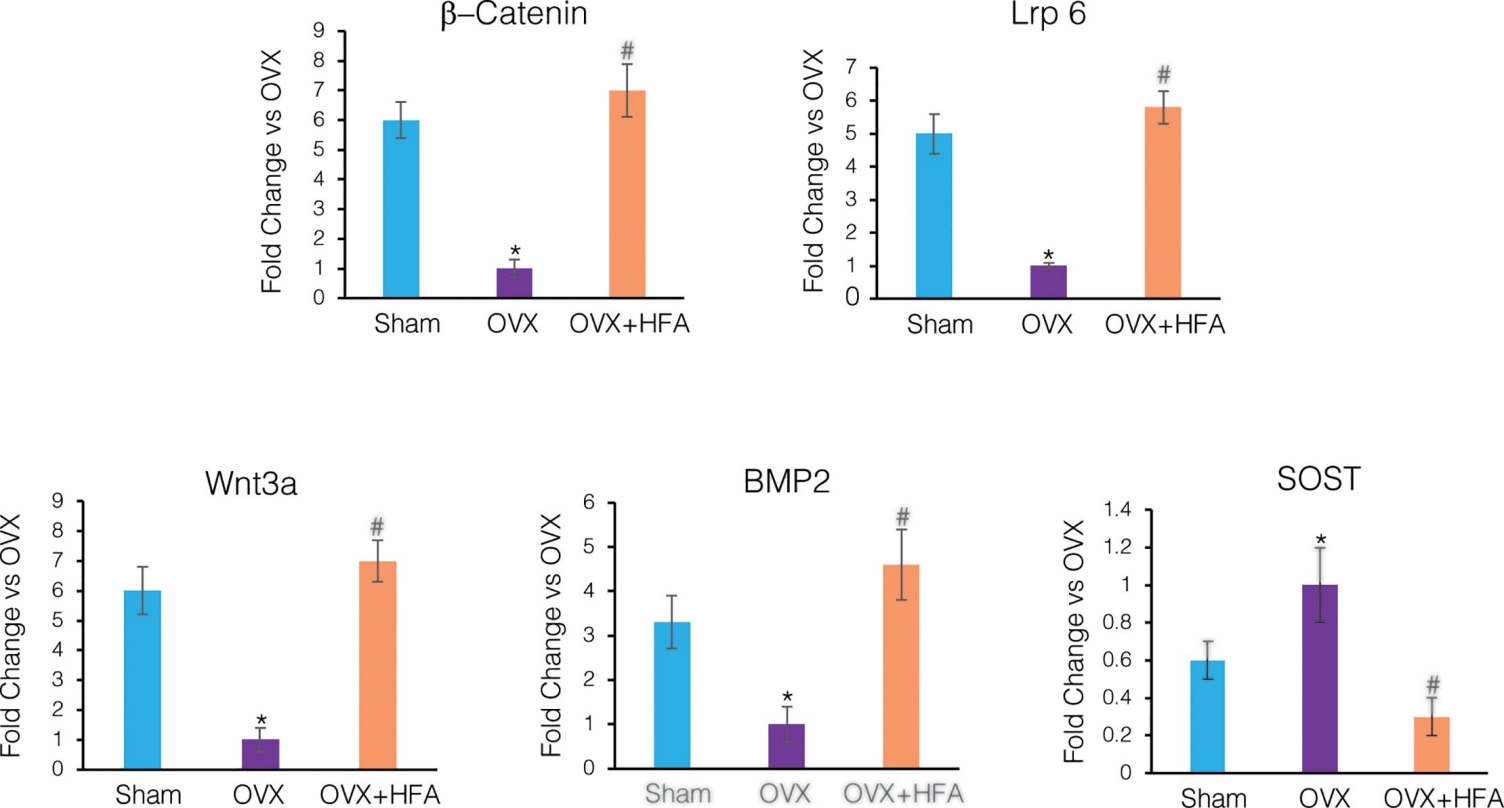
Expression of members of Wnt and BMP2 signaling pathways decreased, while SOST increased, in alveolar bone in response to HFA. Gene expression of Wnt signaling genes (*β*-catenin, Lrp6, Wnt3a), BMP2 and SOST was measured by real time RT-PCR. Data show mean fold change in mRNA levels compared to OVX, 56 days after application of HFA to the maxillary right first molar. Each value represents the mean ± SEM of 6 animals (* Significantly different from Sham group; ^#^ significantly different from OVX group).

To study pathways that may be activated in response to vibration, gene expression levels of the canonical Wnt (Wnt3a, Lrp6, *β*-catenin), the Wnt pathway inhibitor SOST and BMP2 signaling were evaluated (Fig 7). In the OVX group members of the canonical Wnt signaling pathway and BMP2 were down-regulated compared to Sham levels (p<0.05). Application of HFA up-regulated the expression of these genes, which was statistically significant (p<0.05) compared to the OVX group. SOST, which is a negative bone formation regulator, was significantly higher in the OVX group than the Sham group. HFA stimulation for 28 days inhibited the upregulation of SOST expression (p<0.05).

**Fig 8:**
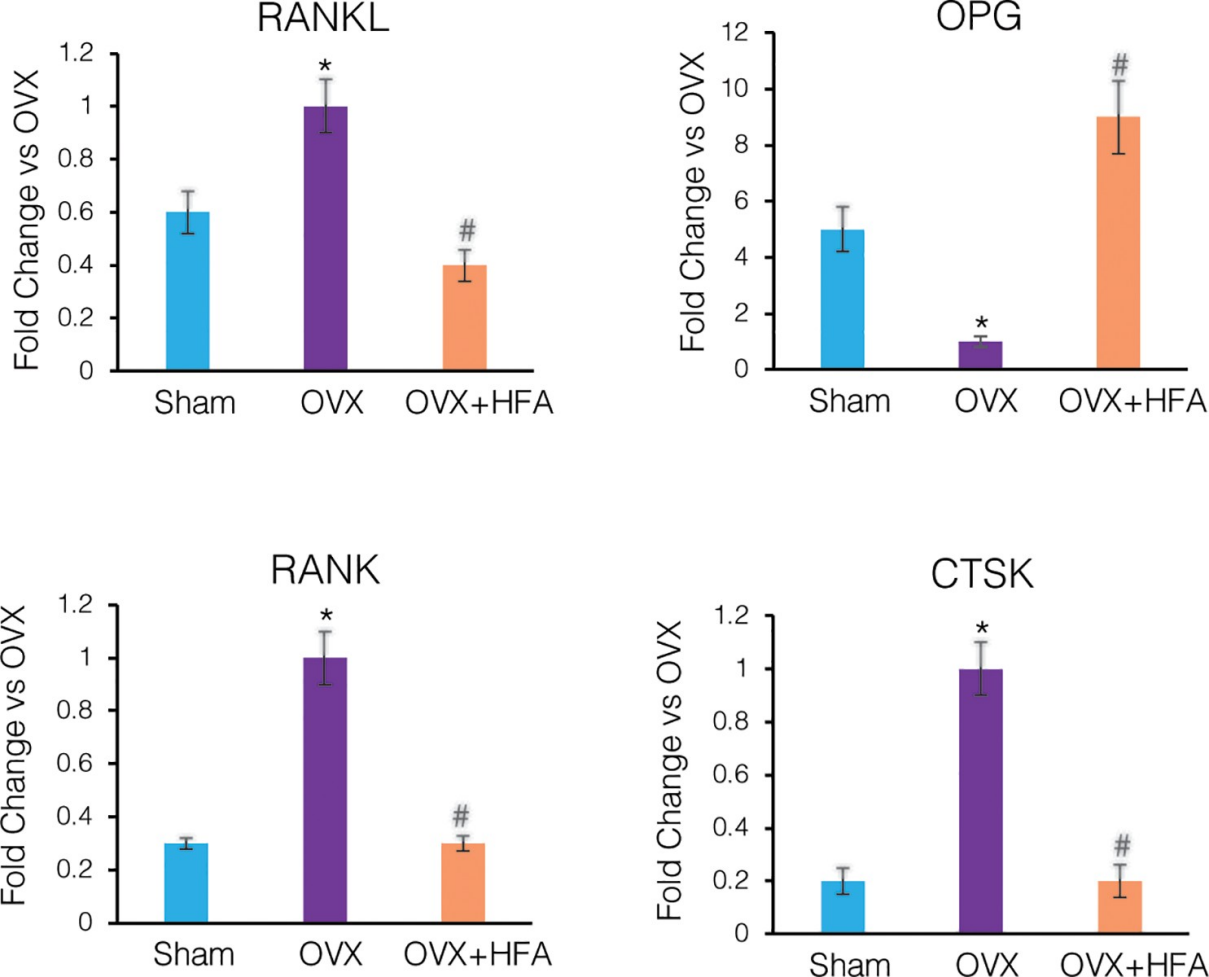
HFA decreased expression of osteoclastic markers in alveolar bone. Gene expression of RANKL, OPG, RANK and CTSK was measured by real time RT-PCR. Data shows mean fold change in mRNA levels compared to OVX, 56 days after application of HFA to the maxillary right first molar. Each value represents the mean ± SEM of 6 animals (* Significantly different from Sham group; ^#^ significantly different from OVX group).

Our results also show that OVX animals exhibited decreased OPG expression and increased RANKL, RANK and CTSK (Cathepsin K) expression compared to the Sham group levels (p<0.05) (Fig 8). HFA significantly increased OPG expression and decreased RANKL and RANK and CTSK expression (p<0.05).

**Fig 9:**
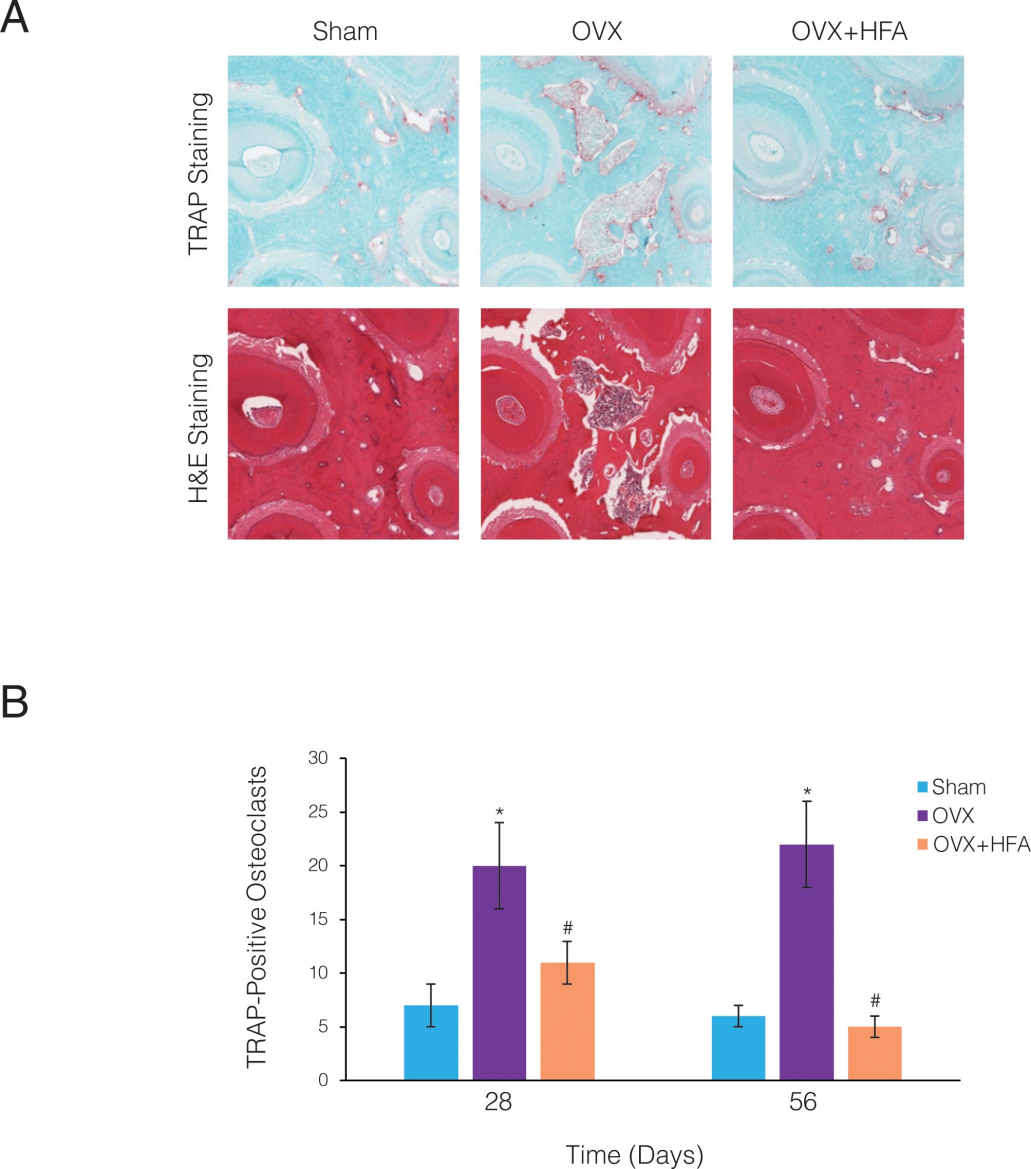
HFA reduced osteoclast numbers in alveolar bone. **(A)** Light microphotographs of H&E and immunohistochemically stained sections for TRAcP-5b. Images were collected close to the mid-root area of the first maxillary molar after 56 days of HFA application. Osteoclasts are identified as TRAcP-5b red cells in sections from different experimental groups. **(B)** Mean number of osteoclasts present in 5 consecutive sections of the ROI of the maxillary first molar. Each value represents the mean ± SEM of 6 animals (* Significantly different from Sham group; # significantly different from OVX group).

To investigate if the increase in the osteoclast differentiation markers leads to increased osteoclastogenesis, osteoclast presence was evaluated as TRAcP-5b positive cells in immunohistochemically stained sections of the alveolar bone surrounding the maxillary first molar (Fig 9A). While osteoclasts were densely clustered in the OVX rats, only sporadic osteoclasts were visible in the Sham bone. The number of TRAcP-5b cells was significantly higher in the OVX than the Sham group at both Day 28 (p<0.015) and Day 56 (p<0.005) (Fig 9B). The OVX+HFA group, however, showed a significant decrease in osteoclast number compared to the OVX group at both Day 28 (p<0.006) and Day 56 (p<0.001).

### Osteogenic effect of high-frequency acceleration was not limited to the point of application and could restore the mechanical properties of alveolar bone

**Fig 10:**
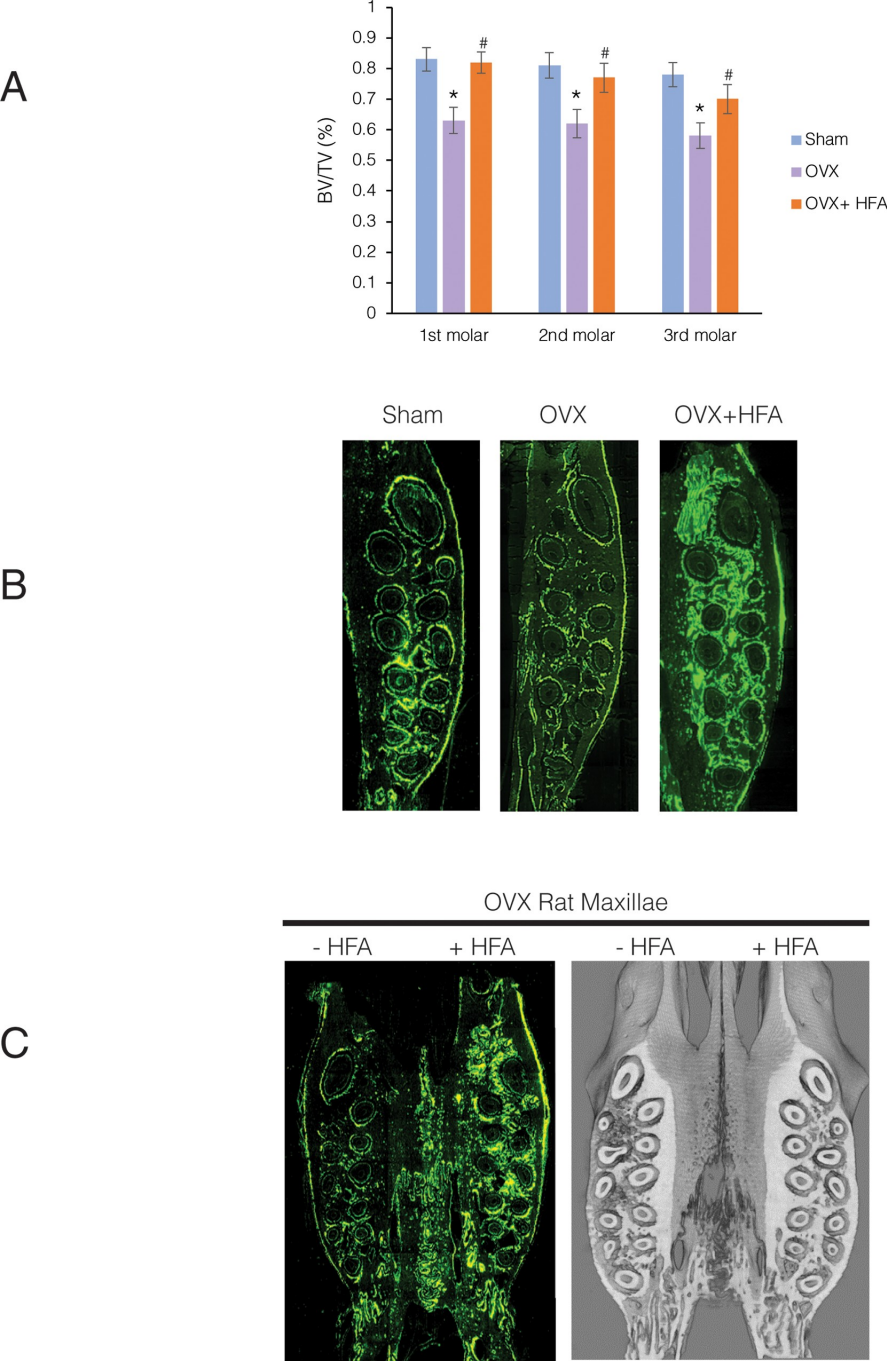
HFA had a gradient effect and restored the mechanical properties of osteoporotic alveolar bone. **(A)** BV/TV was calculated form μCT analysis for inter-radicular bone of maxillary first, second and third molars 56 days after HFA application for Sham, OVX and OVX+HFA groups. Data are expressed as the mean 6 samples ± SEM (* significantly different from the Sham group). **(B)** Rats received demeclocycline (50mg/kg) at Day 0 and Day 54 and calcein green (15mg/kg) at Day 28. Animals were euthanized on Day 56. Fluorescence microscopy of sagittal sections at Day 56 shows increased intensity of the fluorescent labels in OVX+HFA maxillae compared to OVX maxillae, showing extensive bone formation especially at the point of HFA application (red arrow). **(C)** Fluorescence microscopy and μCT images of axial sections of maxillae in animals that received HFA on one side (HFA positive) and not the other side (HFA negative). Note the increase of the osteogenic signal only on the side that received HFA.

To evaluate if the osteogenic effect of HFA was limited to the point of application (the maxillary first molar in our study) or if it can restore bone density in adjacent areas, the effect of HFA on inter-radicular bone of the second and third molars was evaluated. Quantitative analysis of μCT images demonstrates a significant decrease in bone density at each molar in the OVX group compared to the Sham group (p<0.05) (Fig 10A). There was no significant difference in the BV/TV between the first, second and third molars (p>0.05), showing that osteoporosis extended throughout the alveolar bone. Application of HFA to the first molar of OVX+HFA animals not only improved the bone density in the inter-radicular bone of the first molar (by 19%) (p<0.003), but also improved the bone density in the inter-radicular bone of the second molar (by 15%) (p<0.02) and the third molar (by 12%) (p<0.045). These changes were statistically significant compared to the same areas in the OVX group. While no significant differences between inter- radicular bone density of the first, second and third molars in the OVX+HFA group was observed (p>0.05), a gradient effect of HFA was visible with the greatest changes occurring closest to the source of HFA application. Fluorescent microscopy of undecalcified sections of alveolar bone supports this observation by clearly showing that the increase in bone formation activity spread to the adjacent area (Fig 10B).

To determine if HFA applied to the right maxillary alveolar bone affects the alveolar bone density on the left maxillary alveolar bone, the effect of HFA on the contra-lateral hemi-maxillae was studied. No significant osteogenic activity was observed under fluorescent microscopy or μCT analysis on the opposite side of the maxilla, which suggests the effect of HFA is attenuated by intermediate tissues, such as sutures and by the distance (Fig 10C).

**Fig 11:**
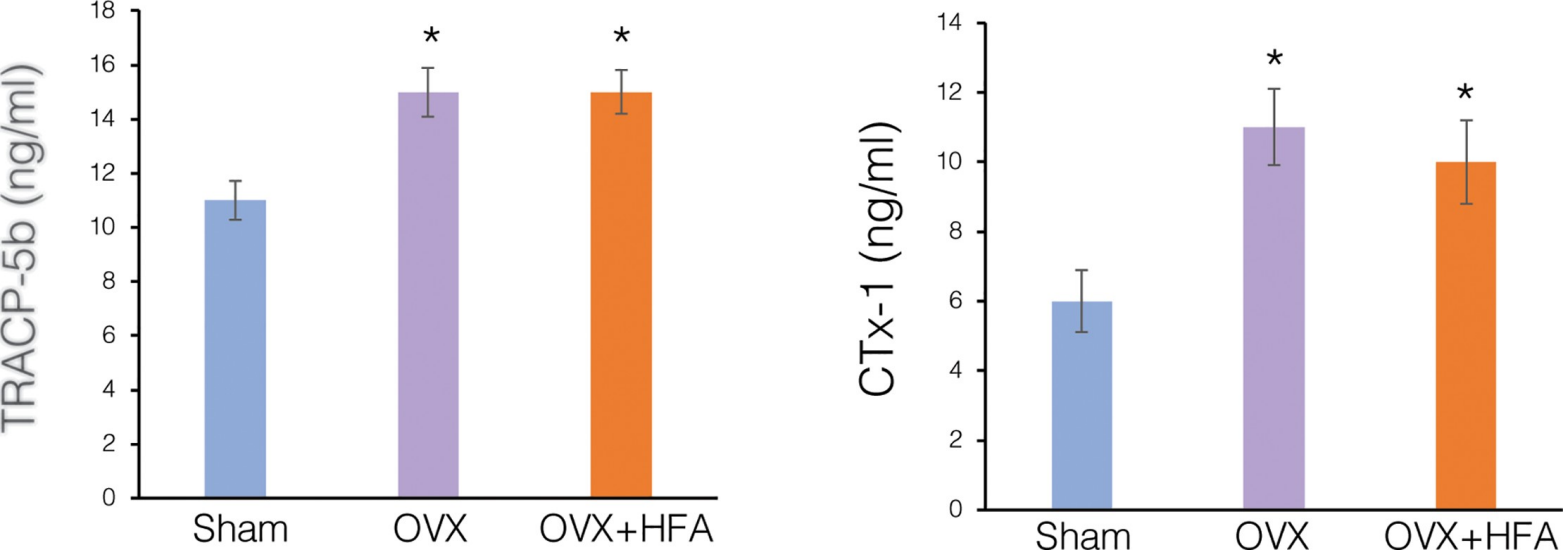
Local application of HFA did not affect systemic markers of bone resorption in OVX rats. Serum levels of Tartrate resistant acid phosphatase-5b (TRAcP-5b) and C terminal telopeptide of type 1 collagen (CTX-1) were measured at Day 56 by ELISA. Data are expressed as the mean of 6 samples ± SEM (* significantly different from Sham group).

To investigate if HFA applied locally has any systemic effect, bone resorption markers tartrate-resistant acid phosphate 5b (TRAcP-5b) and C-terminal telopeptides of type I collagen (CTx-1) were measured at Day 56 in the serum of Sham, OVX and OVX+HFA groups by ELISA (Fig 11). Serum TRAcP-5b and CTx-1 concentrations were significantly higher in the OVX (p<0.006) and OVX+HFA (p<0.005) groups compared to the Sham group. There was no significant difference in the TRAcP-5b and CTx-1 levels between the OVX and OVX+HFA groups (p>0.05).

To evaluate if the increase in bone volume and bone density effects the mechanical properties of the maxilla, a three-point bending test was performed. Compared to the alveolar bone in the Sham group, the alveolar bone of the OVX group showed significantly weaker mechanical properties. The maximum failure load was 14% lower in the OVX group compared to the Sham group, which was statistically significant (p<0.018) (Table 2).

**Table 2:** Three-point bending failure load of the rat maxilla.

	Sham	OVX	OVX+ HFA	*p values	^#^p values
**Maximum Load (N)**	134 ± 8	106 ± 6*	131± 9^#^	p<0.018	p<0.043
**Yield Load (N)**	123 ± 7	103 ± 5*	124 ± 8^#^	p<0.042	p<0.05
**Stiffness (N/mm)**	479±29	388±24*	468±25^#^	p<0.036	p<0.043

Data are presented as the mean ± SEM of 6 samples.

* Significantly different from the Sham group

# significantly different from the OVX group.

HFA treatment caused an approximately 11% increase in failure load compared to the OVX group, which was statistically significant (p<0.043). Similarly, compared to the Sham group, the OVX group showed a 13% and 24% decrease in Yield Strength (p<0.042) and Stiffness (p<0.036), respectively, which were both statistically significant (p<0.05). No statistical differences were observed between the mechanical properties of alveolar bone in Sham and OVX+HFA groups after 56 days (p>0.05).

## Discussion

The current study builds on our previous work demonstrating that local application of vibration in the form of low magnitude load but high frequency acceleration (frequency of 120 Hz, acceleration of 0.3 g and loading of 10 *με*) significantly increases bone density in healthy alveolar bone and maintains the alveolar bone level after extraction [21, 22]. The effect of increasing acceleration and frequency is not linear, and after a certain level, increasing the frequency does not increase the osteogenic effects [24].

We now show that the anabolic effects of HFA are not limited to healthy bones and vibration helped restore the bone density of osteoporotic alveolar bone. This is clinically very significant considering numerous studies that show positive correlations between estrogen deficiency and tooth loss, periodontitis, delayed alveolar bone wound healing and increased severity of residual alveolar ridge resorption [5, 8, 25, 26]. In agreement with these findings, studies that investigated the effect of different pharmaceutical drugs on osteoporosis of weight bearing bones reveal a promising correlation between treatment of osteoporosis and a decrease in the possibility of tooth loss in these patients [27, 28]. However, due to side effects associated with pharmaceutical approaches, non- pharmaceutical treatments such as whole-body vibration are gaining popularity. While whole-body vibration (30hz and 0.3g) improves osteoporosis in weight bearing bones [16, 29], there is no report on the therapeutic effect of this approach on non-weight bearing bone, such as osteoporotic alveolar bone, due primarily to the attenuating effect of soft tissue [30]. To the best of our knowledge, our study is the first to demonstrate the therapeutic effect of local application of vibration in the treatment of osteoporotic alveolar bone in ovariectomized rats.

Ovariectomized rat and osteoporotic human bone share many characteristics. Important for our study, they both demonstrate an increased rate of bone turnover, with resorption exceeding formation. This makes the ovariectomized rat model suitable to study postmenopausal bone loss. In fact, this model is approved as a preclinical model by the Food and Drug Administration (FDA) [31]. However, disagreement does arise regarding the effect of ovariectomy on alveolar bone loss. Some studies show that bone loss following ovariectomy predominantly occurs in endochondral bones, such as weightbearing bones, and does not affect intramembranous bones, such as the craniofacial skeleton [31–33]. On the other hand, other studies demonstrate a positive correlation between osteoporosis and alveolar bone changes in ovariectomized rats. Three factors may account for this variability: 1) the animal’s age; 2) duration of the post-ovariectomy period; 3) the jaw that was examined (upper vs lower). The majority of studies that do not show any effect of ovariectomy on alveolar bone have been conducted less than 12 weeks after ovariectomy [34, 35]. Interestingly, all studies with a post-ovariectomy period longer than 12 weeks report reduced mineral content and structural changes in alveolar bone [36–38]. This can be explained by the delay in the appearance of osteoporosis in non-weight bearing bones versus weight bearing bones [39], which may be due to a difference in the embryonic origin (endochondral vs. intramembranous) of the bones and the mechanical environment that may protect the alveolar bone for a longer time [40]. Due to these findings our study was conducted 12 weeks after ovariectomy.

Considering that trabecular bone is more susceptible to estrogen deficiency, the maxillary bone, especially the porous posterior maxillary region is more suitable to study the effect of osteoporosis than the less porous mandibular alveolar bone, which has been the focus of the majority of studies [32, 33, 40–42]. In our study, we focused on the inter-radicular bone since it is entirely trabecular bone, making it a prime target for osteoporosis. This area is also clinically very important since it represents the type of bone involved in periodontal disease and is the recipient site for dental implants. Similar to previous studies, we demonstrated that the osteoporotic effect of estrogen deficiency is not limited to weight bearing bones and can affect alveolar bone, especially in the maxilla.

Our results confirm previous studies that local vibration activated the sequential expression of osteogenic markers and pathways in alveolar bone. We found an initial expression of the osteogenic transcription factors Foxo1, Runx2, and Osterix, which may play a role in the differentiation of mesenchymal cells toward the osteoblast lineage [43–45]. We next found increased expression of the early osteoblast differentiation markers alkaline phosphatase (ALP) and type I*α*1 collagen (ColI*α*1), and late osteoblast differentiation marker osteocalcin (OC), again in agreement with previous studies [46–48]. Multiple signaling pathways may be involved in mediating the effect of vibration in the activation of these osteogenic biomarkers. One possible pathway is the canonical Wnt pathway.

Wnts are a family of secreted proteins extensively expressed within the skeleton that bind to membrane-bound Frizzled and Lrp5/6 co-receptors. When activated, Frizzled and Lrp5/6 receptors stabilize *β*-catenin in the cytoplasm, which then upregulates the expression of osteogenic biomarkers [49, 50]. Our results show that the gene expression of canonical Wnt signaling pathway members (Wnt3, Lrp6, and *β*-catenin) in hemimaxillae was significantly lower in the OVX group compared to the Sham group, and that local vibration was able to activate this pathway. It should be emphasized that other pathways in addition to the Wnt pathway (such as BMP2 and TGF-*β* signaling pathways) are likely involved in mediating the anabolic effect of local vibration. Our results also showed an increase in BMP2 expression in response to vibration; however, future studies are required to dissect the roles different pathways play in mediating the osteogenic activity in response to vibration.

We found increased serum CTx-1 and TRAcP-5b in the OVX and OVX+HFA groups compared to Sham group. This demonstrates higher bone resorption activity following ovariectomy, which agreed with previous studies [51, 52]. Importantly, we also found that local application of vibration did not decrease these biomarkers in the serum, which suggests that the vibration effect is local and not systemic. This higher resorptive activity can be related to activation of RANKL/RANK signaling in the OVX group. OPG/RANKL/RANK signaling has been identified as an essential pathway in modulating osteoclast differentiation and activation [53]. Our results show up- regulation of RANKL/RANK and down regulation of OPG in the OVX group. Local vibration decreased the level of expression of RANKL/RANK and increased the expression of OPG, which was accompanied with a lower number of osteoclasts in alveolar bone.

Another molecule that may be involved in the anti-catabolic effects of vibration is Sclerostin (SOST), which is a negative regulator of bone formation expressed by osteocytes. Decreased mechanical loads on bone significantly increases SOST expression [54]. SOST also antagonizes Wnt signaling by specifically binding to Lrp5/6 co-receptors. Our results demonstrate that SOST expression was significantly higher in the OVX group, which suggests a lack of sensing and detecting external mechanical signals by osteocytes. Local vibration inhibited increased SOST expression, which confirms previous studies that showed decreased SOST expression in response to whole-body vibration [55, 56].

Based on the three-point bending analysis, our study demonstrated that the OVX groups had significantly decreased maxillary alveolar bone structural properties, including maximum load, stiffness and yield load. Interestingly, all of these parameters were improved in response to application of local vibration, which agreed with previous studies [30, 57].

In our study, ovariectomized rats had increased food intake and body weight, which is consistent with previous reports [58, 59]. It has been shown that application of whole- body vibration partly restores body weight in ovariectomized animals [60, 61]. The reduction of weight gain associated with whole-body vibration has been attributed to the suppression of adipogenesis and promotion of osteoblastogenesis by mesenchymal cells [62, 63]. Our results did not show any reduction in body weight of the OVX group in response to application of local vibration. While application of vibration locally may increase mesenchymal cell differentiation into osteoblasts (as was observed by increased expression of RUNX2, Foxo1 and Osterix transcription factors in alveolar bone), it did not have any systemic effect. This agrees with the observation that vibration applied on one side of the maxilla did not have an effect on the contralateral alveolar bone.

The highest osteogenic effect of HFA was observed closest to the HFA application site, which agrees with previous studies suggesting that the osteogenic effect of mechanical stimulation is site-specific. Interestingly, and confirming our previous studies in healthy alveolar bone [22], the osteogenic effect of HFA showed a gradient response, exhibiting a steadily decreasing anabolic effect away from the point of application, both horizontally and vertically. This is clinically extremely useful, especially in situations when the area to stimulate is too fragile to receive direct HFA application, such as after placement of an implant, an extraction, or other pathologic condition that may have debilitated or reduced the bone density. In these cases, application of HFA directly on the area is not possible and may compromised the healing process. However, the possibility of applying HFA on an adjacent area and taking advantage of the gradient effect of HFA, opens new possibilities in clinical dentistry.

Applying HFA to alveolar bone produces paradoxical effects on bone metabolism, with both anabolic and catabolic responses occurring. Interestingly, these paradoxical effects appear to hinge on metabolic conditions at the time HFA is applied. Healthy [21], post-extraction healing [22] and, now, osteoporotic alveolar bone all have an anabolic response to HFA, while alveolar bone around orthodontically moved teeth has a catabolic response to HFA [24]. We have hypothesized [24] that HFA is anabolic when the initial conditions represent physiologically states of bone remodeling (such as a healing alveolar socket) and the tissue mediating the effect is the bone itself. Our results on HFA-induced reversal of osteoporosis support this hypothesis, as osteoporosis is a physiological bone remodeling response to decreased estrogen. In contrast to these normal physiological bone remodeling responses to HFA is the catabolic response seen in orthodontic tooth movement. In this case, we hypothesized [24] that the target of HFA is the periodontal ligament, where inflammation is highly localized in response to orthodontic force. Although much more research is needed to clarify the mechanism for HFA’s paradoxical effects on alveolar bone, our findings suggest that HFA-induced bone remodeling depends on the inflammatory condition of the tissue. In absence of a sustained inflammatory condition, HFA is anabolic however in presence of inflammatory conditions, catabolic effect of HFA will overcome its anabolic effect. This is not surprising, considering that normal physiologic activity such as exercise (whether a patient has healthy bone or osteoporotic bone) will stimulate bone formation, while similar physical activity during an acute inflammatory stage, such as a bone fracture, will worsen the condition.

Our study clearly establishes HFA stimulation as a non-invasive approach to recover bone density lost due to osteoporosis. This investigation could have an enormous impact in clinical dentistry and on the quality of life for millions of patients with osteoporosis or other debilitating conditions of the jaws.

## Supporting information

S1 Table Composition of the normal AIN-93M diet consisting of 3.5% mineral mixture (modified from Nakada et al., 2011, Journal of Hard Tissue Biology, p107-114). (PDF)

S2 Table Composition of low-mineral diet consisting of the same main components as the normal diet but with Ca, P, Mg, Zn and F reduced in quantity to <0.001% of the original composition (modified from Nakada et al., 2011, Journal of Hard Tissue Biology, p107-114). (PDF)
