# Supplementary material for: Therapeutic effect of localized vibration on alveolar bone of osteoporotic rats
Source: PLoS One. 2019 Jan 29;14(1):e0211004. doi: 10.1371/journal.pone.0211004 (PMC6350965; doi:10.1371/journal.pone.0211004)
Supplement: S1 Table — (PDF) [file pone.0211004.s001.pdf]

S 1 Table: Composition of the normal AIN-93M diet consisting of 3.5% mineral mixture (modified from Nakada et al., 2011, Journal of Hard Tissue Biology , p107-114)

Composition	%
Mineral Mixture	3.5
Casein	$1.4\times10^{+1}$
L-Cystine	1.8×10^{-1}
β - Cornstarch	$4.7\times10^{+1}$
α-Cornstarch	$1.6\times10^{+1}$
Sucrose	$1.0\times10^{+1}$
Soybean Oil	4.0
Cellulose Powder	5.0
Vitamin	1.0
Choline Bitartrate	0.3
Tert-Butylhydroquinone	0.8×10^{-3}

Composition of 3.5% minerals	Minerals (%)
$CaCO_3$	1.2
KH_2PO_4	8.8×10^{-1}
$K_3C_6H_5O_7 \cdot H_2O$	9.8×10^{-2}
NaCl	2.6×10^{-1}
K_2SO_4	1.6×10^{-1}
MgO	8.4×10^{-2}
$FeC_6H_5O_7 \cdot x\ H_2O$	2.1×10^{-2}
$ZnCO_3$	5.8×10^{-3}
$MnCO_3$	2.2×10^{-3}
$CuCO_3 \cdot Cu(OH)_2 \cdot H_2O$	1.1×10^{-3}
KIO_3	3.5×10^{-5}
Na_2SeO_4	3.6×10^{-5}
$(NH_4)Mo_7O_{24} \cdot 4\ H_2O$	2.8×10^{-5}
$Na_2SiO_3 \cdot 9\ H_2O$	5.1×10^{-3}
$CrK(SO_4)_2 \cdot 12\ H_2O$	9.6×10^{-4}
LiCl	6.1×10^{-5}
H_3BO_3	2.9×10^{-4}
NaF	2.2×10^{-4}
$NiCO_3 \cdot 2\ Ni(OH_2) \cdot 4\ H_2O$	1.1×10^{-4}
NH_4VO_3	2.3×10^{-5}
Sucrose	7.3×10^{-1}
